# Postoperative outcomes following pancreaticoduodenectomy: how should age affect clinical practice?

**DOI:** 10.1186/1477-7819-11-131

**Published:** 2013-06-06

**Authors:** Walid Faraj, Raafat Alameddine, Deborah Mukherji, Khaled Musallam, Ali Haydar, Mohamed ELoubiedi, Ali Shamseddine, Ali Halal, Ghassan K Abou-Alfa, Eileen M O’Reilly, Faek Jamali, Mohamed Khalife

**Affiliations:** 1 Liver Transplantation and Hepatopancreaticobiliary Surgery, Department of General Surgery, American University of Beirut, Cairo Street, Beirut 1107 2020, Lebanon; 2 Memoria Sloan-Kettering Cancer Center and Weill Medical College at Cornell University, 300 East 66th Street, New York, NY 10065, USA

## Abstract

**Background:**

Pancreaticoduodenectomy is an increasingly common procedure performed for both benign and malignant disease. There are conflicting data regarding the safety of pancreatic resection in older patients. Potentially modifiable perioperative risk factors to improve outcomes in older patients have yet to be determined.

**Methods:**

The American College of Surgeons National Surgical Quality Improvement Program (ACS NSQIP) database for 2008 to 2009 was used for this retrospective analysis. Patients undergoing pancreaticoduodenectomy were identified and divided into those above and below the age of 65. Preoperative risk factors and postoperative morbidity and mortality were evaluated.

**Results:**

Among 2,045 patients included in this analysis, 994 patients were >65 years (48.6%) while 1,051 were (less than or equal to) 65 years (51.4%). Thirty-day mortality was higher in the older age group compared to the younger age group 3.6% vs. 1.9% respectively, *P* = 0.017, odds ratio 1.94. Older patients had a higher incidence of unplanned intubation, ventilator support >48 h and septic shock compared with younger patients. On multivariate logistic regression, after adjusting for other 30-day postoperative occurrences (significant at the *P* <0.1 level) only septic shock was independently associated with a higher odds of mortality, unplanned intubation, and ventilator support >48 h in older patients compared with younger patients.

**Conclusions:**

This report from a population-based database is the first to highlight postoperative sepsis as an independent risk factor for mortality and morbidity in older patients undergoing pancreatic resection. Careful perioperative management addressing this issue is essential for patients over the age of 65.

## Background

Pancreatic cancer is the fourth leading cause of cancer-related death in the United States with peak incidence between the ages of 60 and 80 years [1].

Pancreaticoduodenectomy or the Whipple procedure is a potentially curative treatment for selected pancreatic and periampullary cancers. Evolution of surgical techniques and improvements in postoperative care have contributed substantially to its rising popularity [2]. Over the past three decades, mortality rates following pancreaticoduodenectomy for benign and malignant disease have dropped to less than 2% in high-volume centers and morbidity rates to around 30% [3]. Despite advances in adjuvant therapy, the five-year survival rate following pancreaticoduodenectomy for pancreatic cancer remains low at 15 to 25% [4].

As the general population is aging, a larger segment of the population will be expected to present with conditions requiring the Whipple procedure, thus posing a challenge for surgeons and health care institutions. In the absence of a uniform cutoff age, identifying older patients at higher risk is cumbersome. Delineating the relative contribution of patient baseline risk factors, operative course and postoperative care to final patient outcomes in older patients is more difficult. While studies from small centers investigating this question lack statistical power, high-volume center studies are flawed by patient selection.

Management of cancer in older patients has become a more pressing clinical concern, with increasingly longer life expectancy. Seventy-five percent of patients with pancreatic cancer are older than 60 years [5]. Currently, age alone is not a contraindication to pancreatic resection [6].

The aim of this study is to compare postoperative outcomes between older (>65 years) and young patients (≤65 years) undergoing pancreaticoduodenectomy, and to identify risk factors that contribute to the observed differences.

## Methods

This was a retrospective cohort study using data from the American College of Surgeons National Surgical Quality Improvement Program (ACS NSQIP) database. Details of the ACS NSQIP (www.acsnsqip.org) have been recently described [7]. The ACS NSQIP is a validated outcomes registry designed to provide feedback to member hospitals on 30-day risk-adjusted surgical mortality and morbidity [8,9]. The database includes de-identified data on perioperative variables and 30-day postoperative outcomes for adult patients undergoing major surgery in participating nonveterans’ administration hospitals. Transplant and trauma cases are excluded, specifically patients who are admitted to the hospital with acute trauma and have surgery for that trauma; any operation done after the patient has been discharged from the trauma stay is included. Trained surgical clinical reviewers collect patient data upon admission from the medical chart, operative log, anesthesia record, interviews with the surgical attending, and telephone interviews with the patient [8]. Data quality is ensured through comprehensive training of the nurse reviewers, an inter-rater reliability audit of participating sites, regular conference calls, and an annual meeting [10].

For this study, the available ACS NSQIP participant use files of the years 2008 (271,368 patients from 211 sites) and 2009 (336,190 patients from 237 sites) were retrieved for major surgeries performed at participating ACS NSQIP medical centers. We identified all Whipple cases using the Current Procedural Terminology (CPT) code: 48150. A total of 2,045 patients were identified and included in this study. In accordance with the American University of Beirut’s guidelines (which follow the US Code of Federal Regulations for the Protection of Human Subjects), institutional review board approval was not needed or sought for our analysis because data were collected as part of a quality assurance activity.

### Risk factors

We divided patients into two groups according to age: more and less than 65 years. The cutoff age of 65 was based on the World Health Organization (WHO) classification for older people. For each patient, data were collected for demographics, preoperative medical history, surgical settings, total operative time, and intraoperative use of packed red blood cells (pRBC) transfusions.

### Outcomes

Evaluated postoperative outcomes were 30-day mortality and morbidity including: [1] wound (deep incisional surgical site infection, organ or space surgical site infection, or wound dehiscence); [2] respiratory (pneumonia, unplanned intubation, or ventilator support >48 h); [3] cardiac (myocardial infarction or cardiac arrest requiring cardiopulmonary resuscitation); [4] cerebrovascular accident; [5] renal (acute renal failure or progressive renal insufficiency; [6] systemic sepsis (sepsis or septic shock); [7] venous thromboembolism (VTE) (deep venous thrombosis or pulmonary embolism); [8] major bleeding requiring >4 units of pRBC within 72 hours; and [9] unplanned return to the operating room (Table 1).

**Table 1:** Incidence of 30-day postoperative outcomes in young and older patients undergoing the Whipple procedure

**Outcome**	**Young**	**Older**	***P *value**	**OR (95% CI)^a^**
	**n = 1051**	**n= 994**		
**MORTALITY**	20 (1.9)	36 (3.6)	0.017	1.94 (1.11-3.37)
**MORBIDITY**				
**Wound**	154 (14.7)	149 (15.0)	0.830	1.03 (0.81-1.31)
Deep incisional SSI	27 (2.6)	29 (2.9)	0.629	1.14 (0.67-1.94)
Organ/space incisional SSI	122 (11.6)	110 (11.1)	0.700	0.95 (0.72-1.25)
Wound dehiscence	17 (1.6)	24 (2.4)	0.199	1.51 (0.80-2.82)
**Respiratory**	99 (9.4)	124 (12.5)	0.027	1.37 (1.04-1.81)
Pneumonia	53 (5.0)	65 (6.5)	0.147	1.32 (0.91-1.91)
Unplanned intubation	48 (4.6)	69 (6.9)	0.021	1.56 (1.07-2.28)
Ventilator support >48 h	58 (5.5)	77 (7.7)	0.043	1.44 (1.01-2.05)
**Cardiac**	13 (1.2)	22 (2.2)	0.089	1.81 (0.91-3.61)
Myocardial infarction	3 (0.3)	9 (0.9)	0.067	3.19 (0.86-11.82)
Cardiac arrest necessitating CPR	10 (1.0)	16 (1.6)	0.184	1.70 (0.77-3.77)
**Renal**	21 (2.0)	25 (2.5)	0.431	1.27 (0.70-2.28)
Acute renal failure	11 (1.0)	8 (0.8)	0.569	0.77 (0.31-1.92)
Progressive renal insufficiency	14 (1.3)	17 (1.7)	0.484	1.29 (0.63-2.63)
**Venous thromboembolism**	30 (2.9)	38 (3.8)	0.222	1.35 (0.83-2.20)
Deep vein thrombosis	23 (2.2)	23 (2.3)	0.848	1.06 (0.59-1.90)
Pulmonary embolism	11 (1.0)	15 (1.5)	0.351	1.45 (0.66-3.17)
**Systemic sepsis**	156 (14.8)	159 (16.0)	0.470	1.09 (0.86-1.39)
Sepsis	130 (12.4)	102 (10.3)	0.133	0.81 (0.62-1.07)
Septic shock	33 (3.1)	61 (6.1)	0.001	2.03 (1.31-3.11)
**Cerebrovascular accident**	2 (0.2)	2 (0.2)	0.955	1.06 (0.15-7.52)
**Major bleeding**^**b**^	19 (1.8)	14 (1.4)	0.474	0.78 (0.39-1.56)
**Return to operating room**	91 (8.7)	82 (8.2)	0.740	0.95 (0.69-1.30)

Data presented as n (%).

^a^Odds of outcome in patients >65 years compared with ≤65 years (referent group).

^b^Requiring >4 units of packed red blood cells within first 72 h postoperatively.

*OR* odds ratio, *CI* confidence interval, *SSI* surgical site infection, *CPR* cardiopulmonary resuscitation.

### Statistical analysis

Descriptive statistics are presented as means ± standard deviation (SD), medians (interquartile range (IQR)), or percentages. Risk factors and outcomes were compared between the two age groups (young vs. older) using the chi-squared test for categorical variables and the independent samples *t*-test for continuous variables. The primary study outcome measure was the incidence of mortality in the older group compared with the young group. The secondary study outcome measure was the incidence of any of the nine morbidities in the older group compared with the young group.

To further scrutinize the association between age and postoperative outcomes, we constructed multivariate logistic regression models, where any significant association between age and 30-day mortality or morbidity was adjusted for the occurrence of other 30-day postoperative morbidities significant at the *P* <0.1 level on bivariate analysis. Observing modification of the effect estimates (odds ratios (OR) and 95% confidence intervals (CI)) allowed for the identification of the independent association between age and the postoperative outcome, irrespective of the occurrence of other outcomes.

We also constructed multivariate logistic regression models to identify the modifying effect of risk factors on the estimate of any independent association between age and outcomes. Risk factors were selected if they were different between young and older patients at the *P* <0.1 level. Through adjusting for each risk factor, we were able to determine if it explains the observed association between age and outcome. All *P* values were two-sided with the level of significance set at <0.05.

## Results

Data from 2,045 patients were included in this analysis. Their mean age was 64.1 ± 12.3 years (range: 17 to 90 years), with 52.3% being males. A total of 994 patients were >65 years (48.6%, older group) while 1,051 were ≤65 years (51.4%, young group). Table 2 summarizes the top 10 most common diagnoses in patients undergoing the Whipple procedure from both age groups.

**Table 2:** Top 10 most common diagnosis in young and older patients undergoing the Whipple procedure

**Young (n = 1051)**	**n (%)**
Malignant neoplasm of head of pancreas	304 (28.9)
Malignant neoplasm of pancreas part unspecified	108 (10.3)
Chronic pancreatitis	73 (6.9)
Malignant neoplasm of ampulla of Vater	57 (5.4)
Malignant neoplasm of duodenum	56 (5.3)
Malignant neoplasm of pancreas	53 (5.0)
Benign neoplasm of pancreas except islets of Langerhans	45 (4.3)
Malignant neoplasm of other specified sites of pancreas	37 (3.5)
Unspecified disease of pancreas	27 (2.6)
Malignant neoplasm of extrahepatic bile ducts	24 (2.3)
**Older (n = 994)**	**n (%)**
Malignant neoplasm of head of pancreas	338 (34.0)
Malignant neoplasm of pancreas part unspecified	119 (12.0)
Malignant neoplasm of ampulla of Vater	92 (9.3)
Malignant neoplasm of pancreas	65 (6.5)
Malignant neoplasm of duodenum	48 (4.8)
Benign neoplasm of pancreas except islets of Langerhans	43 (4.3)
Malignant neoplasm of other specified sites of pancreas	35 (3.5)
Malignant neoplasm of extrahepatic bile ducts	25 (2.5)
Malignant neoplasm of pancreatic duct	19 (1.9)
Unspecified disease of pancreas	15 (1.5)

### Risk factors

Older patients undergoing the Whipple procedure were more likely to be at a higher American Society of Anesthesiologists (ASA) class and nonindependent in functional status compared with young patients (Table 3). They were also more likely to have a history of diabetes, hypertension, peripheral vascular disease, transient ischemic attacks, percutaneous coronary intervention, cardiac surgery, and dyspnea at rest or moderate exertion; yet less likely to be obese. Older patients also had a higher prevalence of chronic obstructive pulmonary disease; although they were less likely to be current smokers, within one year of surgery. Both preoperative anemia and intraoperative pRBC transfusions were more common in older compared with young patients.

**Table 3:** Prevalence of risk factors in young and older patients undergoing the Whipple procedure

**Parameter**	**Young**	**Older**	***P *value**
	**n = 1051**	**n = 994**	
Male	552 (52.5)	518 (52.1)	0.853
White race	812 (77.3)	834 (83.9)	<0.001
Totally or partially dependent in functional status	23 (2.2)	39 (3.9)	0.022
ASA classification^a^			<0.001
*I or II*	378 (36.0)	234 (23.5)	
*III*	640 (60.9)	688 (69.2)	
*IV or V*	33 (3.1)	72 (7.2)	
BMI ≥30 kg/m^2^	303 (28.8)	210 (21.1)	<0.001
Diabetic on oral agents or insulin	213 (20.3)	262 (26.4)	0.001
Alcohol intake in two weeks prior (>2 drinks/day)	46 (4.4)	28 (2.8)	0.059
Hypertension requiring medication	412 (39.2)	655 (65.9)	<0.001
Dyspnea on moderate exertion/at rest	72 (6.9)	107 (10.8)	0.002
Congestive heart failure in 30 days prior	2 (0.2)	5 (0.5)	0.226
Angina in 30 days prior	3 (0.3)	7 (0.7)	0.175
Myocardial infarction in 6 months prior	4 (0.4)	3 (0.3)	0.760
Previous percutaneous coronary intervention	50 (4.8)	98 (9.9)	<0.001
Previous cardiac surgery	30 (2.9)	93 (9.4)	<0.001
History of peripheral vascular disease^b^	9 (0.9)	21 (2.1)	0.018
Current smoker (within 1 year)	348 (33.1)	140 (14.1)	<0.001
History of severe COPD	42 (4.0)	77 (7.7)	<0.001
History of transient ischemic attack	8 (0.8)	42 (4.2)	<0.001
History of CVA without neuro deficit	18 (1.7)	26 (2.6)	0.160
History of CVA with neuro deficit	12 (1.1)	21 (2.1)	0.082
Anemia^c^	433 (41.2)	511 (51.4)	<0.001
Bleeding disorder	24 (2.3)	36 (3.6)	0.073
Weight loss >10% in 6 months prior	217 (20.6)	190 (19.1)	0.386
Disseminated cancer	32 (3.0)	26 (2.6)	0.559
Tumor involving central nervous system	2 (0.2)	0 (0.0)	0.169
Chemotherapy in 30 days prior	28 (2.7)	15 (1.5)	0.069
Radiotherapy in 90 days prior	37 (3.5)	31 (3.1)	0.613
Ascites in 30 days prior	3 (0.3)	6 (0.6)	0.277
Esophageal varices in 6 months prior	1 (0.1)	0 (0.0)	0.331
Currently on dialysis	3 (0.3)	5 (0.5)	0.431
Prior operation within 30 days	16 (1.5)	24 (2.4)	0.145
Infected surgical wound class	15 (1.4)	19 (1.9)	0.392
Total operation time, min (mean ± SD)	398.9 ± 145.3	369.6 ± 125.1	<0.001
Intraoperative pRBC units transfused			0.001
0	738 (70.2)	620 (62.4)	
1 or 2	193 (18.4)	234 (23.5)	
≥3	120 (11.4)	140 (14.1)	

Data presented as n (%) unless otherwise indicated.

^a^American Society of Anesthesiologists (ASA) scores: I, healthy patient; II, mild systemic disease but no functional limitations; III, severe systemic disease with definite functional limitations; IV, severe systemic disease that is a constant threat to life; and V, moribund patient unlikely to survive 24 hours with or without operation.

^b^Requiring revascularization, angioplasty, or amputation.

^c^Hematocrit concentration <36% in women and <39% in men. BMI, body mass index.

*COPD* chronic obstructive pulmonary disease, *CVA* cerebrovascular accident, *SD* standard deviation, *pRBC* packed red blood cell.

### Postoperative outcomes

Mortality rate was found to be higher in the older age group (3.6% vs. 1.9% young, *P* = 0.017, OR 1.94 (95% CI 1.11 to 3.37). Older patients had a higher incidence of unplanned intubation, ventilator support >48 h and septic shock compared with younger patients. However, on multivariate logistic regression analysis and after adjusting for other 30-day postoperative occurrence (significant at the *P* <0.1 level) age was only independently associated with a higher odds of septic shock, with the latter association explaining the higher odds of mortality, unplanned intubation, and ventilator support >48 h in older compared with younger patients (Table 4).

**Table 4:** Adjustment of significant associations between age and 30-day postoperative outcomes for the incidence of other postoperative outcomes

**Factor**	**30-day postoperative outcome in older vs. young patients**			
	**Mortality**	**Unplanned intubation**	**Ventilator support >48 h**	**Septic shock**
**UNADJUSTED**	1.94 (1.11-3.37)	1.56 (1.07-2.28)	1.44 (1.01-2.05)	2.03 (1.31-3.11)
**ADJUSTED FOR**				
Myocardial infarction	1.89 (1.08-3.29)	1.51 (1.03-2.21)	1.42 (0.99-2.01)	1.97 (1.28-3.05)
Unplanned intubation	1.63 (0.89-2.96)		1.21 (0.79-1.85)	1.84 (1.11-3.07)
Ventilator support >48 h	1.77 (1.01-3.12)	1.39 (0.88-2.19)		1.92 (1.16-3.19)
Septic shock	1.56 (0.88-2.80)	1.16 (0.74-1.82)	1.08 (0.71-1.64)	

The median duration to the incidence of septic shock in the older patient group was 8 days (IQR: 4 to 12 days, min: 1 day, max: 29 days) which was similar to the younger patient group (median: 8 days, IQR: 3.5 to 13 days, min: same day, max: 28 days). After adjusting for all risk factors that had a different prevalence between younger and older patients (significant at the *P* <0.1 level), the odds ratio for septic shock in older compared with younger patients remained essentially unchanged; indicating that none of the evaluated risk factors could explain the observed association between age and septic shock (Table 5).

**Table 5:** Association between age and 30-day postoperative septic shock upon adjustment for risk factors

**Factor**	**Septic shock**		
	**Young**	**Older**	***P *value**
	**OR (95% CI)**	**OR (95% CI)**	
**UNADJUSTED**	Referent	2.03 (1.31-3.11)	0.001
**ADJUSTED FOR**			
White race	Referent	2.00 (1.30-3.09)	0.002
Totally or partially dependent in functional status	Referent	1.94 (1.26-3.00)	0.003
ASA classification	Referent	1.77 (1.14-2.75)	0.010
BMI ≥30 kg/m^2^	Referent	2.06 (1.34-3.19)	0.001
Diabetic on oral agents or insulin	Referent	2.01 (1.30-3.10)	0.002
Alcohol intake in two weeks prior (>2 drinks/day)	Referent	2.04 (1.32-3.14)	0.001
Hypertension requiring medication	Referent	1.84 (1.18-2.89)	0.007
Dyspnea on moderate exertion/at rest	Referent	1.91 (1.23-2.95)	0.004
Previous percutaneous coronary intervention	Referent	1.94 (1.25-2.99)	0.003
Previous cardiac surgery	Referent	1.90 (1.22-2.94)	0.004
History of peripheral vascular disease	Referent	2.00 (1.30-3.10)	0.002
Current smoker (within 1 year)	Referent	2.09 (1.34-3.26)	0.001
History of severe COPD	Referent	1.94 (1.25-2.99)	0.003
History of transient ischemic attack	Referent	1.99 (1.28-3.07)	0.002
History of CVA with neuro deficit	Referent	2.01 (1.31-3.10)	0.002
Anemia	Referent	1.95 (1.26-3.00)	0.003
Bleeding disorder	Referent	1.95 (1.26-3.02)	0.003
Chemotherapy in 30 days prior	Referent	2.05 (1.33-3.16)	0.001
Total operation time	Referent	2.12 (1.37-3.28)	0.001
Intraoperative pRBC transfusion	Referent	1.88 (0.76-1.87)	0.005

*OR* odds ratio, *CI* confidence interval, *ASA* American Society of Anesthesiologists, *BMI* body mass index, *COPD* chronic obstructive pulmonary disease, *CVA* cerebrovascular accident, *pRBC* packed red blood cell.

## Discussion

In this large multicenter study, we have found that patients over the age of 65 undergoing pancreaticoduodenectomy have increased postoperative mortality rates compared to younger patients largely due to an increased risk of septic shock. Despite the fact that older patients had a higher prevalence of pre- and intraoperative interoperative risk factors, these could not explain the observed increase in the incidence of septic shock in this cohort. Our findings have important clinical implications for management of the older patient undergoing pancreatic resection.

We abandoned the classical look at the chronological age as an absolute cutoff splitting patients into safe and danger zones [11,12]. We used age 65 as proposed by WHO to look deeper into the inherent characteristics of older patients [6]. This cutoff is closer to the mean age in our analysis (64.1 years), providing us with the highest statistical power to come to robust conclusions. We included a comprehensive list of risk factors associated with older age and studied their effect on postoperative outcomes using multivariate analysis.

When looking at the indications for pancreatic resection, we found that malignant processes whether in older or in young patients, account for the largest group of performed procedures (Table 2). Tumors at the head of the pancreas account for only 62.9% of cases, reflecting the widening distribution of indications as well a rising acquaintance by surgeons. Notably, chronic pancreatitis, while being the third diagnosis in the young age group, does not appear among the most common 10 diagnoses in the older age group. This finding is likely to reflect a persistent hesitance in performance of the Whipple procedure for benign conditions in older patients rather than decreased incidence in this cohort. We have found that patients over the age of 65 undergoing pancreaticoduodenectomy have increased postoperative mortality rates compared to younger patients largely due to an increased risk of septic shock. In previous reports, the incidence of postoperative septic shock had been overlooked. Postoperative mortality was associated with the pathology of the underlying resection specimen [13,14], intraoperative blood loss [15], postoperative surgical complications [16,17], advanced ASA score, history of dyspnea [5], or preoperative hypoalbuminemia [18].

Old age, defined as more than 65 years, is an established risk factor for developing sepsis regardless of other comorbidities [19]. Patients in this age category also fare worse in terms of survival or late-term morbidity following septic shock [20]. Several hypotheses attempted to explain this association. Aging of the immune system ‘immunosenescence’ attributes this observation to the decreased capacity of the immune system to properly handle foreign microorganisms with advanced age [21,22]. Functional decline in innate as well as adaptive immune systems have been described in animal models and human cell lines and correlated with decreased immune function [23-27]. Beside failure of development of adequate defensive response against foreign pathogens, aging is also associated with prolonged release of inflammatory cytokines. Aged mice cells were more affected by the levels of lipopolysaccharide (LPS) and tumor necrosis factor (TNF) than younger cells [28]. An exaggerated response to inflammatory markers was also found to exist in older patients [29,30]. Low levels of albumin and total cholesterol, common in older patients, are other surrogate markers for susceptibility to increased sepsis and mortality [31-33]. The syndrome of frailty affecting older people has recently received special attention. Underlying sarcopenia is a generalized catabolic state with a general decline of physiological body reserves. A frailty index has been developed and found to predict postoperative outcomes in older patients [34].

The strong correlation between septic shock and mortality draws attention to the contribution of postoperative care in optimizing outcomes in older patients. The fact that age by itself is a risk factor for the development of septic shock mandates a rapid response to early sign of infection. Aggressive intervention is of utmost importance to prevent rapid deterioration of older patients. These data call for further investigation of antibiotic prophylaxis for older patients undergoing pancreatic resection.

Limitations of this study include lack of information regarding antibiotic treatment in this cohort. Since the ACS NSQIP does not include data on the use of antimicrobial agents, so we could not evaluate the association between the use of certain drugs and the incidence of septic shock. Another potential limitation of this study was that we were unable to control for hospital effects owing to the absence of hospital identifiers in our data. There may have been variability in hospital quality or variability in surgical strategy, which may have potentially confounded the association between risk factors and outcome. Finally, the possibility of omitted variable bias is always present in observational studies.

Our study provides a new insight to the understanding of postoperative complications following pancreatic resection. It sheds light on the increased risk of sepsis in older patients. It also emphasizes the importance of postoperative care as equal to the patient baseline risk profile and intraoperative course in ensuring safety of outcomes. Interestingly, the contribution of septic shock was masked in previous single institution studies. Therefore, inter-institutional differences in postoperative care are major contributors to the mortality and morbidity of patients’ post-Whipple procedure. Further research should be focused on improving prophylactic measures to prevent and early identify sepsis among older patients.

We suggest the following protocol for those patients: in the preoperative setting, a multidisciplinary approach is performed for each patient.

A thorough cardiac, pulmonary and anesthetic evaluation is performed. Presurgical biliary drainage is performed for patients presenting with severe jaundice (total bilirubin more than 5 mg/dl, normal range is 0 to 1.2). Older patients are admitted 24 to 48 hours prior to surgery. Preoperative subcutaneous anticoagulation according to weight is given the night before surgery. All patients will be on intravenous antibiotics using broad-spectrum coverage for multi organisms including Gram-positive, Gram-negative, anaerobes and fungal infections. Patients are educated for using the incentive spirometer 24 to 48 hours prior to surgery. Intraoperative monitoring with repetitive blood testing is routinely performed. Blood transfusion is only given when the hemoglobin is less than 8 g/dl (normal range is 12 to 18 g/dl).

Most of our patients are transferred to a regular ward postoperatively, only critical patients are transferred to the intensive care unit.

Postoperative antibiotics are maintained for 3 to 4 days, anticoagulation therapy is maintained until the time of discharge.

## Conclusions

In conclusion, age is not a contraindication to pancreatic resection, however, this study highlights the fact that older patients have increased morbidity and mortality related to increased risk of postoperative sepsis. With this information, protocols for older patients to minimize these increased risks should be developed.

## Competing interests

The authors declare that they have no competing interests.

## Authors' contributions

WF: First author and Main designer, RA: second author, Helped in the main design, DM: Editing of the manuscript, KM: Data analysis, AH: Editing of the manuscript, ME: Data analysis, AS: Senior consultant for the design of the manuscript, AH: Data collection, GA: Senior consultant for the design of the manuscript, EO: Senior consultant for the design of the manuscript, FJ: Data Collection, MK: Senior author and consultant for the design of the manuscript. All authors have read and approved the final manuscript.
